# Analysis of sleep problem in children aged 1–3 years with autism spectrum disorder in Zhejiang province, China

**DOI:** 10.3389/fpsyt.2022.923757

**Published:** 2022-08-30

**Authors:** Dan Yao, Shasha Wang, Fangfang Li, Minjie Gao, Jie Shao

**Affiliations:** Department of Pediatric Health Care, The Children's Hospital, Zhejiang University School of Medicine, National Clinical Research Center for Child Health, Hangzhou, China

**Keywords:** autism spectrum disorder, children, sleep status, sleep problems, quality, China

## Abstract

**Background:**

High prevalence of sleep problems have been founded in children with Autism Spectrum Disorder (ASD), with rates ranging from 50 to 80%. We aimed to study the sleep status and the occurrence of sleep problems in children with autism spectrum disorder (ASD) aged 1–3 years, and to provide reference for guiding early comprehensive intervention for ASD children from the perspective of sleep.

**Methods:**

From January 1 to December 31, 2021, 74 ASD children who met the diagnostic criteria of “Diagnostic and Statistical Manual of Mental Disorders-5 (DSM-V)” served as case group while 84 typically-developing children of the same sex and age served as control group. An original Children's Sleep Habit Questionnaire was adopted to compare the sleep status of children in the two groups and to conduct statistical analysis on related factors.

**Results:**

The incidence of sleep problems in the case group (78.4%) was significantly higher than that in the control group (34.5%) (*P* < 0.001). Compared with the children in the control group, children in the case group had later bedtime (*P* < 0.05) and less sleep duration (*P* < 0.05), and required longer time to fall asleep (*P* < 0.001) The incidence of sleep problems in children who could fall asleep autonomously in the case group was significantly lower than that in children who needed parental help (*P* < 0.05). In the case group, the longer the screen exposure time, the higher the incidence of sleep problems (*P* < 0.05).

**Conclusions:**

The incidence of sleep problems in ASD children aged 1–3 years is also high, mainly manifested in late bedtime, difficulty falling asleep, frequent night awakenings and less sleep duration. Both sleep patterns and screen exposure can impact their sleep. In the early comprehensive intervention of ASD children, it is necessary to pay full heed to their sleep status and take timely intervention measures in order to improve the quality of life for the ASD children and their families.

## Introduction

Autism spectrum disorder (ASD) is a chronic, lifelong neurodevelopmental disability characterized by impairments in social communication, restricted interests or activities, and repetitive behaviors (1). In the last decade, the prevalence rate of ASD has showed a sharp rise (2). The prevalence rate announced in the United States in 2006 was 0.9%, and the reported prevalence rate in 2020 has reached 1.8% (3). In China, the prevalence rate has rose from 0.35% in 2018 (4) to 0.7% in 2020 (5). This may be due to the advances in our understanding of the etiology of autism and the improved diagnostic techniques. ASD is a life-long disorder that has major implications for the quality of life of the individual with ASD as well as the families, resulting in a tremendous responsibility on the part of the public health and education systems. The comorbidity of ASD, such as sleep problems, gastrointestinal problems, and attention deficit hyperactivity disorder, has attracted more and more attention (6, 7). Studies have reported that the incidence of sleep problems in ASD children is 50–80% (8–10), much higher than the 29–50% reported in typically-developing children with normal development (11, 12). Sleep as an important physiological function impacts many aspects of early childhood development (13). For ASD children, sleep problems not only exacerbate certain core symptoms, such as restricted and repetitive behaviors and problematic behaviors, but may also affect the effectiveness of rehabilitation training (14–16). Previous studies have found that the sleep problems in ASD children are age-specific (12). But these research reports both at home and abroad aim at all preschool children and school-age children (17, 18). At present, the sleep status of ASD children aged 1–3 years has not been reported at home and abroad, and the impact of sleep problems on infants is far greater than that of children or adults. During the infant period, sleep occupies most of the time in their life. Good sleep not only contributes to the maturation of the central nervous system and the formation of the overall function of the infant, but also has an important influence on the formation of physical, cognitive, neuromotor and temperament development (19). Therefore, this study finds out the sleep characteristics of ASD children aged 1–3 years by analyzing the occurrence of sleep problems and sleep status, and provides a reference for early comprehensive intervention and improvement of their quality of life.

## Materials and methods

### Participants

Case group: 77 children with a diagnosis of ASD by the Children's Hospital, Zhejiang University School of Medicine from January 1 to December 31, 2021. Inclusion criteria: (1) Aged 1–3 years; (2) The diagnosis of ASD was performed independently by a developmental pediatrician and a psychiatrist using the DSM- 5 (20) in combination with the ADOS-2 (21, 22); (3) Excluding other organic diseases, hearing disorders, neurodevelopmental disorders, genetic metabolism diseases as well as mental and psychological disorders; (4) Stable medical conditions, and not taking psychiatric or sleep related medications in the past 3 months. A total of 77 questionnaires were collected, of which 74 (62 males and 12 females) were valid. The dropout rate was 3.9%.

Control group: 86 typically-developing children who underwent physical examinations in the same period. Inclusion criteria: (1) Aged 1–3 years; (2) Conforming to the developmental process of children of the same age through routine examination by the Children's Hospital, Zhejiang University School of Medicine; (3) Excluding other organic diseases, hearing disorders, neurodevelopmental disorders, genetic metabolic diseases as well as mental and psychological disorders; (4) Stable medical conditions, and not taking psychiatric or sleep related medications in the past 3 months. A total of 86 questionnaires were collected, of which 84 (65 males and 19 females) were valid. The dropout rate was 2.3%.

This research was approved by The Children's Hospital, Zhejiang University School of Medicine and Affiliation of Ethics Committee, and informed consent was obtained from the guardians of the research subjects.

### Sample size

The sample size calculation used the formula of two independent sample rates:


$$n1=n2=\frac{{\left[Z_{\alpha /2}\sqrt{2\bar{p}\left(1-\bar{p}\right)}+Z_{\beta }\sqrt{p_{1}\left(1-p_{1}\right)+p_{2}\left(1-p_{2}\right)}\right]}^{2}}{{\left(p_{1}-p_{2}\right)}^{2}}\;$$

It was reported that the highest prevalence of sleep problems in the ASD was 80% (10), while that in typically-developing children with normal development was 50%. Alpha was set to 0.05, beta was 0.1, and missing rate was 10%. The sample size required for each group was 57 in each centers through calculation.

### Original children's sleep habit questionnaire

As Children's Sleep Habit Questionnaire (CSHQ) is mainly used to assess the sleep status of children aged 4–10 (23, 24), its factor structure is not applicable for young children. Therefore, an original Children's Sleep Habit Questionnaire was used in this research based on to the sleep characteristics of children aged 1–3 years, which mainly included: children's basic information, general family information, children's bedtime, sleep latency, sleep duration at night, sleep duration in the day time, sleep patterns, difficulty falling asleep, night awakening, parasomnia (such as sleepwalking, teeth grinding), sleep-disordered breathing (such as snoring, dyspnea during sleep) and sleep behavior, etc. This original Children's Sleep Habit Questionnaire was reviewed and validated by several rounds of focus group interviews of experts in the relevant field of child health care. It was done in Mandarin Chinese. The language validation was checked, reviewed and revised by the experts during the focus group interviews in terms of its readability and understandability. This questionnaire should be filled in by parents based on their children's sleep status in the last month. The sleep problems is defined as children with difficulties in falling asleep, frequent night awakening (more than once a night), sleep-disordered breathing, parasomnia at least 3 times a week for at least one month according to the common sleep problems of ASD children and the sleep characteristics of healthy children aged 1–3 years.

### Quality control

The questionnaire and informed consent are issued by professionals who have undergone unified training, and the content and precautions of the scales are explained by use of unified guidelines. The questionnaire should be filled out by the primary caregiver of the child. After the completion of the filling, it is reviewed by professionals for on-site supplement or correction of any omission, unclear information, and incorrect filling.

### Statistical analysis

The SPSS26.0 statistical software was used to analyze the data. The experimental data of this study were qualitative, expressed as percentages (%). Comparison between groups was performed by sample Chi-square test. Trend comparison of bedtime was performed by The Mann—Whitney test. The difference was statistically significant with *P* < 0.05.

## Results

### Overview of the research subjects

A total of 74 children (62 males, 12 females; mean age 30.62 ± 4.43 months) with a diagnosis of ASD and 84 healthy controls (65 males, 18 females; mean age of 30.90 ± 4.49 months) of the same age and sex were enrolled. No statistical significance in age and sex ratio existed between the two groups of children (*P* > 0.05), indicating comparability (Table 1).

**Table 1:** Demographical factors of the case group and the control group.

**Cases**	**Case group**	**Control group**
Total	74	84
1–2year	12 (16.2%)	14 (16.7%)
>2–3year	62 (83.8%)	60 (83.3%)

### Comparison of the incidence of sleep problems between the case group and the control group

The incidence of sleep problems in the case group was 78.4%, significantly higher than that in the control group (34.5%) (*P* < 0.001). The incidence of difficulty falling asleep (48.6%) was significantly higher than that of the control group (22.6%) (*P* = 0.001), and the incidence of frequent night awakenings (24.2%) was also obviously higher than that of the control group (9.5%) (*P* < 0.05). No statistical significance in the incidence of sleep-disordered breathing and parasomnia existed between the two groups (*P* > 0.05) (Table 2).

**Table 2:** Comparison of the incidence of sleep problems between the case group and the control group.

**Sleep problems**	**Case group**	**Control group**	***P***
**(case%)**	**(*n =* 74)**	**(*n =* 84)**	
Total	58 (78.4%)	29 (34.5%)	0.000
Difficulty falling asleep	36 (48.6 %)	18 (22.6%)	0.001
Frequent night awakenings	18 (24.2%)	8 (9.5%)	0.011
Sleep-disordered breathing	7 (9.5%)	6 (7.1%)	0.404
Parasomnia	4 (5.4%)	5 (6.0%)	0.579

### Comparison of sleep status between case group and control group

Statistical significance in the distribution of bedtime existed between the two groups, and the trend of bedtime was also statistically different (*P* < 0.001), indicating that the time for children to go to bed at night in the case group was obviously later than that in the control group. Compared with the control group, the children in the case group took significantly longer time to fall asleep (*P* < 0.001), and the sleep duration at night was less than that in the control group (*P* < 0.05). No statistically significant difference in awakening time in the morning and sleep duration in the daytime existed between the two groups (*P* > 0.05) (Table 3).

**Table 3:** Comparison of sleep status between case group and control group.

**Item**		**Case group (*n =* 74)**	**Control group (*n =* 84)**	***P***
	Before 9:00	4 (5.4%)	20 (23.8%)	
Bedtime	9:00–10:00	34 (45.9%)	42 (50%)	0.001
	10:00–11:00	24 (32.4%)	18 (21.4%)	
	After 11:00	12 (16.2%)	4 (4.8%)	
	<30 min	14 (18.9%)	66 (78.6%)	
Sleep latency	30 min−1 h	34 (45.9%)	18 (21.4%)	0.000
	1 h−2 h	22 (29.7%)	0 (0%)	
	>2 h	4 (5.4%)	0 (0%)	
	Before 7:00	19 (25.7%)	36 (42.9%)	
Morning wake-up time	7:00–8:00	37 (50.0%)	28 (33.3%)	0.111
	8:00–9:00	16 (21.6%)	18 (21.4%)	
	After 9:00	2 (2.7%)	2 (2.4%)	
Sleep duration at night	<10 h	40 (54.1%)	27 (32.1%)	0.005
	≥10 h	34 (45.9%)	57 (67.9%)	
	No nap	2 (2.7%)	0 (0.00)	
Sleep duration in the day time	0–1 h	6 (8.1%)	11 (13.1%)	0.117
	1–2 h	39 (52.7%)	32 (39.3%)	
	>2 h	27(36.5%)	40 (47.6%)	

### Comparison of the incidence of sleep problems between the two groups of children under different sleep behaviors and screen exposure

For the sleep pattern, the proportion of children who could fall asleep autonomously in the case group was significantly lower than that of the children in the control group, and the difference between the two groups had statistical significance (*P* = 0.001). In the control group, the incidence of sleep problems in children who could fall asleep autonomously was significantly lower than that in children who needed parental help (such as breastfeeding, hugging) (*P* < 0.05). The influence of whether the patient could fall asleep autonomously on the sleep problem was more significant in the case group than in the control group, and the difference had statistical significance (*P* < 0.001) (Tables 4, 5).

**Table 4:** Comparison of different sleep behaviors and screen exposure between case group and control group.

**Item**		**Case group (*n =* 74)**	**Control group (*n =* 84)**	***P***
Sleep pattern	Fall asleep autonomously	24 (32.4%)	49 (58.3%)	0.001
	Fall asleep with parental help	50 (67.6%)	35 (41.7%)	
Screen exposure	<1 h	24 (32.4%)	36 (42.9%)	0.178
	≥1 h	50 (67.6%)	48 (57.1%)	
Parent-child interaction	<1 h	24 (32.4%)	37 (44.0%)	0.135
	≥1 h	50 (67.6%)	47 (56.0%)	

**Table 5:** Comparison of the incidence of sleep problems between the two groups of children under different sleep behaviors and screen exposure.

	**Item**		**Cases**	**Sleep problem (%)**	***P***
Case group	Sleep pattern	Fall asleep autonomously	24	13 (54.2%)	<0.001
		Fall asleep with parental help	50	45 (90%)	
	Screen exposure	<1 h	24	12 (50%)	0.025
		≥1 h	50	38 (76%)	
	Parent-child interaction	<1 h	24	18 (75%))	0.418
		≥1 h	50	40 (80%)	
Control group	Sleep pattern	Fall asleep autonomously	49	7 (14.6%)	0.021
		Fall asleep with parental help	35	13 (36.1%)	
	Screen exposure	<1 h	36	8 (22.2%)	0.488
		≥1 h	48	12 (25%)	
	Parent-child interaction	<1 h	37	7 (18.9%)	0.478
		≥1 h	47	13 (21.7%)	

There was no statistical difference in the duration of screen exposure time existed between the case and the control groups (*P* > 0.05). In the case group, the longer the screen exposure time, the higher the incidence of sleep problems (*P* < 0.05), while the children in the control group had no statistical difference in the duration of screen exposure and the occurrence of sleep problems (*P* > 0.05) (Tables 4, 5).

There was no statistical difference in the duration of parent-child interaction between the case group and the control group, also no statistical in the duration of parent-child interaction and the occurrence of sleep problems in each group (*P* > 0.05) (Tables 4, 5).

## Discussion

Children with ASD are extremely vulnerable to sleep problems due to underlying biological and behavioral rhythms that predispose them to extrinsic and intrinsic stressors that affect sleep (25). Sleep disorders may adversely affect children's daily function, thus affecting behavior, learning, memory regulation and cognition (26), and may also cause emotional problems such as aggression, irritability, over-reactivity and depression (27). Other studies have found that core symptoms also affect sleep, such as communication difficulties may exacerbate sleep disorders (28). All these problems not only reduce the quality of life, but also influence the effect of intervention. These connections all illustrate the severity of sleep problems in ASD. There is growing evidence that ASD is associated with arousal dysregulation and sensory hyper-reactivity, and calming strategies may be helpful in improving sleep (29).

### Occurrence of sleep problems in ASD children aged 1–3 years

Researches have shown that children with neurodevelopmental disorders are more susceptible to sleep problems (30, 31), and ASD children have the highest incidence of 50–80% (8, 9). In this research, the incidence of sleep problems in the ASD children was 78.4%, significantly higher than that in the control group (34.5%), which was consistent with the conclusion given by Inthikoot N, et al. who found that the incidence of sleep problems in school-aged children with ASD was higher than that in typically-developing children (32). It was also higher than the incidence (67.4%) of sleep problems in ASD children aged 2–7 who underwent a large-scale multi-center study from 2018 to 2019 (33), showing that ASD children before the age of 3 also had sleep problems, and the incidence may be higher.

ASD children often suffer from comorbid sleep disorders mainly manifested as frequent night awakenings, difficulty falling asleep, late sleep habits, night terrors, excessive early awakening, short sleep duration, circadian rhythm disturbance, daytime sleepiness, sleep-disordered breathing, etc., (27). The etiology and pathogenesis are still unclear, but may be related to the influence of social environment, neurodevelopmental function, comorbidities, intestinal diseases, and respiratory diseases. It is generally believed that the interaction of biological and social or environmental factors lead to neurosecretory dysfunction and changes in sleep habits (34). A multi-center study by Hongyu Chen et al. found that the four dimensions with the highest prevalence of sleep problems in ASD children aged 2–7 were bedtime resistance (25.6%), sleep anxiety (22.7%), sleep delay (17.9%) and daytime somnolence (14.7%) (33). This study showed that difficulty falling asleep and frequent night awakenings were the most common sleep problems in ASD children aged 1–3 years with the incidence of 48.6 and 24.2%, respectively, which was obviously higher than that of the control group. Sleep-disordered breathing accounted for a small percentage of sleep problems, supporting previous findings of negligible relationship between breathing difficulties and ASD (35, 36). No statistical difference in sleep-disordered breathing and parasomnia existed between the case group and the control group. It can be seen that the manifestations of sleep problems vary in different age groups, which is determined by the sleep characteristics of different age groups. ASD children are usually excited before going to bed and cannot calm down quickly after going to bed, so they are difficult to fall asleep. These sleep problems make it even harder to foster an ASD child. For this reason, ASD children should be helped to establish good sleeping habits from an early age and create a quiet and comfortable sleeping environment. Before going to bed, they shall be provided with regular preparatory activities, and improved sleep procedures, and shall be prevented from strenuous exercise, overeating, watching cartoons so as to improve sleep.

### Description of sleep status of ASD children aged 1–3 years

A growing number of researches have shown that sleep problems have a negative impact on the daily lives of ASD children (37), and will exacerbate social communication problems and repetitive behaviors (38). Sikora et al. found that sleep problems were negatively correlated with daytime behaviors in 1,193 ASD children, and ASD children had more internalized behavior problems and poorer adaptability. Children with moderate or severe sleep problems had more behavioral problems than children with mild sleep problems (39). The guidelines issued by the WHO in 2019 define sleep duration <10 h a day is insufficient sleep duration for preschoolers (40). This study showed that ASD children had later bedtime and less sleep duration, and longer time to fall asleep autonomously than that in the control group. No statistically significant difference in awakening time in the morning and sleep duration in the daytime existed between the two groups. This is consistent with the results of a study by Miano S et al. that 22.6% of children with ASDs slept for fewer than 8 h a night compared with only 9.6% of typically developing children (41). It is of great significance to timely master the sleep status of ASD children and provide targeted interventions.

### Effects of different sleep patterns and parenting styles on sleep quality in ASD children aged 1–3 years

Previous studies have shown that the sleep pattern affects the occurrence of sleep problems (42, 43). This study has shown that a significant difference in sleep patterns exists between the case group and the control group, and children in the case group are more difficult to sleep autonomously. Nevertheless, the incidence of sleep problems in children who could fall asleep autonomously in both groups was lower than that in children who need parental help. Whether, ASD children could fall asleep autonomously in the case group had a more obvious impact on sleep problems than that in the control group. It can be seen that children who need help to fall asleep, such as family members' hugging, cuddling, nursing, rely on other people's support. They have a high incidence of sleep problems due to poor transition ability from mild awakening to sleep (44), and ASD children are more vulnerable. Therefore, we need to begin from the cultivation of autonomous sleep from childhood in order to improve the sleep quality of ASD children.

Screen exposure usually refers to the contact and use of electronic products such as mobile phones, computers, and TVs. With further development of scientific research, the adverse effects of screen exposure on children's sleep have also attracted the attention of scholars worldwide (45, 46). The guidelines issued by the American Academy of Pediatrics in 2016 pointed out that electronic products should not be used by children under the age of 1.5 years, and screen exposure time for children aged 2–5 years should not be exceed 1 h a day (47). This study found that screen exposure of both the case group and the control group was long, but no statistical difference existed between the two groups. More than half of children aged 1–3 years have more than an hour of screen time a day, which also reflected a bad current situation in China. Yet, with the same screen exposure, the longer the screen exposure time, the higher the incidence of sleep problems in the case group, while there is no significant difference between screen exposure time and sleep problems in the control group. It can be seen that ASD children aged 1–3 years with more screen exposure are more susceptible to sleep problems, while the exposure factor has little impact on sleep quality for typically-developing children. This will also provide a new guideline for sleep problems in ASD children aged 1–3 years. Reduced screen exposure needs to be incorporated into sleep guidelines for children with autism.

## Conclusion

Our study described and discussed the sleep problems and related factors of ASD children aged 1–3 years in detail, so as to provide information for the comprehensive diagnosis and intervention of ASD children in the younger age group. The sleep status of ASD children is closely related to the pathography of autism. Moreover, ASD children aged 1–3 years have later bedtime, less sleep duration at night, difficulty falling asleep, and frequent night awakenings, which made it even harder to foster an ASD child for the guardians. We must pay sufficient attention to the sleep status of ASD children aged 1–3 years. At the same time, cultivating autonomous sleep and reducing screen exposure time can improve sleep quality of ASD children to some extent, and are also worthy of our attention when behavioral intervention improves sleep quality. In summary, sleep is an important guarantee for children's neurodevelopment and maturation. Parents and medical staff are called upon to detect sleep problems in ASD children early and conduct behavioral interventions timely in order to improve the quality of lives for ASD children and their families (48).

## Limitations

The sample age of this study is limited to ASD children aged 1–3 years, so the results of this study cannot be extended to ASD patient over 3 years old. Meanwhile, this study did not analyze the intelligence level and comorbidities of the subjects. So further research is needed in this aspect. In addition, This study is essentialy based on subjective reports from parents. Objective assessment (i.e., with actigraphy or polisomnography) can be carried out in the future for in-depth research on the sleep problems of ASD children.

## Data availability statement

The original contributions presented in the study are included in the article/supplementary material, further inquiries can be directed to the corresponding author.

## Ethics statement

The studies involving human participants were reviewed and approved by the Ethics Committee of the Children's Hospital, Zhejiang University School of Medicine. Written informed consent to participate in this study was provided by the participants' legal guardian/next of kin.

## Author contributions

DY conceptualized and designed the study, enrolled patients, interpreted the data, drafted the initial manuscript, and revised the manuscript. SW enrolled patients, collated and inputted the data, conducted the analysis, and interpreted the data. FL and MG collected and reviewed the questionnaire. JS conceptualized and designed the study and reviewed and revised the manuscript for important intellectual content. All authors approved the final content of the manuscript.

## Funding

This research was supported by grants from Natural Science Foundation of Zhejiang Province (LQ20H090017 to DY).

## Conflict of interest

The authors declare that the research was conducted in the absence of any commercial or financial relationships that could be construed as a potential conflict of interest.

## Publisher's note

All claims expressed in this article are solely those of the authors and do not necessarily represent those of their affiliated organizations, or those of the publisher, the editors and the reviewers. Any product that may be evaluated in this article, or claim that may be made by its manufacturer, is not guaranteed or endorsed by the publisher.
